# The Prevalence and Extent of Molar-Incisor Hypo-Mineralization by Gender in a Group of Iranian Children

**DOI:** 10.18502/ijph.v49i8.3911

**Published:** 2020-08

**Authors:** Zahra BAHROLOLOOMI, Narjes AMROLLAHI, Nasrin MOSTAFALOO

**Affiliations:** 1. Oral Health Research Center, Department of Pediatric Dentistry, School of Dentistry, Shahid Sadoughi University of Medical Sciences, Yazd, Iran; 2. Dental Research Center, Department of Pediatric Dentistry, School of Dentistry, Isfahan University of Medical Sciences, Isfahan, Iran; 3. Department of Pediatric Dentistry, School of Dentistry, Semnan University of Medical Sciences, Semnan, Iran

## Dear Editor-in-Chief

Molar-Incisor Hypo-mineralization (MIH) is defined as dysplasia of enamel structure caused by defects in ameloblasts during early developmental stages. Clinically, defect in enamel translucency is interpreted as demarcated opacity of white, cream, yellow or brown colors (1, 2). Toothache, increased sensitivity, malformation, increased susceptibility to plaque accumulation are considered as complications of MIH (3, 4). The definitive cause of molar-incisor hypoplasia is unknown, but it can be related to various environmental factors associated with systemic conditions (1, 5).

We aimed to determine the prevalence of MIH by gender in 7–11-year-old children in the city of Yazd, Iran.

The present cross-sectional descriptive-analytical study was approved by the Ethics Committee of Shahid Sadooghi University of Medical Sciences in Yazd (Code no: 173).

Overall, 645 children of 7 to 11 yr old were selected from schools in Yazd using a randomized cluster sampling method, and examined by the same person using a dental explorer and mirror. Children with amelogenesis imperfect, fluorosis, or color change due to tetracycline and children with smaller than 1 mm defects were excluded. Severity and extent of MIH lesions were measured using modified Developmental Defect Enamel (mDDE) and European Academy of Pediatric Dentistry (EAPD) criteria(1), and its relation with gender was assessed. Based on EAPD criterion, teeth with only white or yellow-brown color change were regarded as mild, those appearing with enamel loss as moderate, and teeth with affected dentin as severe. This criterion categorizes lesions in terms of extent into three groups: lesions ≥ 4.5 mm are considered as large, 3.5 mm lesions as medium, and 2 mm lesions as small. mDDE criterion divides MIH lesions according to color and spread of lesion into demarcated opacity, diffuse opacity, and hypoplasia. Parents were asked about their children history of systemic diseases. Mean data were analyzed using the Chi-square test at significance level of *P*<0.05.

Of the 645 children examined, 154 (23.8%) had MIH. In both sexes, molars were equally more affected than incisors. The results showed no significant correlation between MIH and type of tooth affected (mandibular or maxillary molars, and mandibular or maxillary incisors) by gender (*P*=0.538) (Table 1). There was a significant correlation between extent and severity and size of MIH lesions by gender (*P*<0.001). No significant relation was found between gender and color or spread of hypoplastic MIH lesions (*P*=0.103). Molar-incisor hypoplasia >4.5 mm and affecting two-thirds or more of the teeth was significantly more observed in girls (*P*<0.001).

**Table 1:** Prevalence of MIH by gender

***Prevalence of Hypomineralization by gender***		***Girls (%)***	***Boys (%)***	***Total (%)***	***P-value***
Lesion size	<2.5mm	61(25.3)	72(36.7)	133(30.4)	0.001
	2.5–3.5mm	29(12.0)	36(18.4)	65(14.9)	
	>4.5mm	151(62.7)	88(44.9)	239(54.7)	
Severity	Mild	109(45.2)	137(69.9)	246(56.3)	0.000
	Moderate	40(16.6)	23(11.7)	63(14.4)	
	Severe	92(38.2)	36(18.4)	128(29.3)	
Extent of affected teeth	<1.3	75(31.1)	86(43.9)	161(36.8)	0.000
	1.3–2.3	51(21.2)	54(27.6)	105(24.0)	
	>2.3	115(47.7)	56(28.6)	171(39.1)	
Type teeth affected	Mandibular incisor	12(5.0)	8(4.1)	20(4.6)	0.538
	Mandibular molar	104(43.2)	73(37.2)	177(40.5)	
	Maxillary incisor	82(34.0)	78(39.8)	160(36.6)	
	Maxillary molar	43(17.8)	37(18.9)	80(18.3)	
Color and spread of lesions	Demarcated opacity	90(37.3)	93(47.4)	183(41.9)	0.103
	Diffuse opacity	77(32.0)	52(26.5)	129(29.5)	
	Hypo-plastic	74(30.7)	51(26.0)	125(28.6)	

In both sexes, the most common lesions were mild, larger than 4.5 mm lesions, and demarcated opacity form. In all severities, molar-incisor hypoplasia occurred significantly more in girls (*P*<0.001). A review of history of children with MIH provided by parents showed that diarrhea and high fever were the most common diseases affected these children (Table 2).

**Table 2:** Prevalence of diseases in children with MIH

***Disease***	***Children with MIH (%)***	***Disease***	***Children with MIH (%)***
Severe fever	13(8.44)	Seizure	5(3.24)
Diarrhea	18(11.68)	Otitis media	5(3.24)
Asthma	2(1.29)	Urinary infection	6(3.89)
Chicken pox	2(1.29)	Septic sore throat	6(3.89)
Allergy	2(1.29)	History of hospitalization	5(3.24)
Heart problem	1(0.64)	History of specific medications	6(3.89)

Molar-incisor hypo-mineralization has a relatively high prevalence in Yazd children. Severity, size and extent of lesions were greater in girls. The difference in the results from various studies (2, 4) regarding the effect of gender on MIH can be attributed to the difference in sample size and also in chronological development of teeth in two sexes. Moreover, greater severity of lesions in girls can be due to their different oral hygiene behaviors. The study population consisted of 7–11-year-old children from Yazd, it appears that long term mild localized stimuli have led to the incidence of demarcated hypo-plastic lesions and relatively large size of ≥4.5 mm, witch stronger effects in girls than in boys. On the other hand, various diseases and systemic factors may have a role in the incidence of MIH, but MIH cannot be attributed to a definitive cause, and further studies are needed to determine causes in different societies.
